# DSCAM-AS1 Long Non-Coding RNA Exerts Oncogenic Functions in Endometrial Adenocarcinoma via Activation of a Tumor-Promoting Transcriptome Profile

**DOI:** 10.3390/biomedicines10071727

**Published:** 2022-07-18

**Authors:** Oliver Treeck, Florian Weber, Juergen Fritsch, Maciej Skrzypczak, Susanne Schüler-Toprak, Christa Buechler, Olaf Ortmann

**Affiliations:** 1 Department of Gynecology and Obstetrics, University Medical Center Regensburg, 93053 Regensburg, Germany; sschueler@caritasstjosef.de (S.S.-T.); oortmann@caritasstjosef.de (O.O.); 2 Department of Pathology, University Medical Center Regensburg, 93053 Regensburg, Germany; florian.weber@klinik.uni-regensburg.de; 3 Department of Infection Prevention and Infectious Diseases, University Medical Center Regensburg, 93053 Regensburg, Germany; juergen.fritsch@klinik.uni-regensburg.de; 4 Second Department of Gynecology, Medical University of Lublin, 20-090 Lublin, Poland; skrzypm@yahoo.co.uk; 5 Department of Internal Medicine I, University Medical Center Regensburg, 93053 Regensburg, Germany; christa.buechler@klinik.uni-regensburg.de

**Keywords:** endometrial adenocarcinoma, long non-coding RNA, DSCAM-AS1, transcriptome

## Abstract

Accumulating evidence suggests that lncRNA DSCAM-AS1 acts tumor-promoting in various cancer entities. In breast cancer, DSCAM-AS1 was shown to be the lncRNA being most responsive to induction by estrogen receptor α (ERα). In this study, we examined the function of DSCAM-AS1 in endometrial adenocarcinoma using in silico and different in vitro approaches. Initial analysis of open-source data revealed DSCAM-AS1 overexpression in endometrial cancer (EC) (*p* < 0.01) and a significant association with shorter overall survival of EC patients (HR = 1.78, *p* < 0.01). In EC, DSCAM-AS1 was associated with endometrial tumor promotor gene *PRL* and with expression of ERα and its target genes *TFF1* and *PGR*. Silencing of this lncRNA by RNAi in two EC cell lines was more efficient in ERα-negative HEC-1B cells and reduced their growth and the expression of proliferation activators like *NOTCH1, PTK2* and *EGR1*. DSCAM-AS1 knockdown triggered an anti-tumoral transcriptome response as revealed by Affymetrix microarray analysis, emerging from down-regulation of tumor-promoting genes and induction of tumor-suppressive networks. Finally, several genes regulated upon DSCAM-AS1 silencing in vitro were found to be inversely correlated with this lncRNA in EC tissues. This study clearly suggests an oncogenic function of DSCAM-AS1 in endometrial adenocarcinoma via activation of a tumor-promoting transcriptome profile.

## 1. Introduction

In the western countries, the most common malignancy of the female genital tract is endometrial cancer (EC). Behind ovarian and cervical cancer, EC is the third most common cause of gynecologic cancer death in women [1]. There are two types of EC: type I EC, also referred to as endometrioid endometrial carcinoma, is the most frequent type (80%) and is accompanied by increased estrogen blood levels. It develops from hyperplastic endometrial tissue and often exhibits loss of tumor suppressor *PTEN* in 37−61% of all cases [2]. Type I tumors generally consist of better-differentiated cells and tend to have a good prognosis, with a recurrence rate of about 20%. In contrast, type 2 endometrial cancer only develops in the post-menopausal period and is divided in subgroups like serous EC (10–15% of all cases) or clear cell carcinoma (about 5%) [3]). In type 2 EC, receptor HER2 is often overexpressed, whereas E-cadherin is lost; both events are known to promote tumor progression [4].

Important physiological and pathological biological processes including tumorigenesis are not only determined by protein expression. Genomic analyses have shown that although 85% of the human genome is transcribed, only about 2% of these RNAs finally are translated into proteins. The large number of transcripts not coding for proteins suggested that these RNAs might have a more diverse and larger role in biological processes than expected [5]. Non-classical functional RNAs are the stable circular RNAs (circRNAs), long non-coding RNAs (lncRNAs) and the short types of non-coding RNAs like micro RNAs (miRNAs). The function of miRNAs has been examined extensively, identifying them as important regulators of gene expression. In contrast, the function of lncRNAs has not been fully elucidated, particularly in cancer [6]. Previous studies showed that lncRNAs work through various mechanisms and can function as natural antisense transcripts or as miRNA sponges and can also interact with or remodel chromatin. Finally, lncRNAs are able to function at the transcriptional, post-transcriptional and epigenetic level, all resulting in gene regulation [7]. Currently, lncRNA function has come into focus, but further attempts are necessary to elucidate their role in various diseases, including cancer. In recent years, the interest in the cancer-related functions of lncRNAs has significantly increased, including studies on endometrial cancer (reviewed in [8]). Recently, lncRNA expression patterns were identified for molecular-based classification of type I endometrial cancers [9,10].

We examined the lncRNA DSCAM-AS1 (DSCAM Antisense RNA 1), a 1.6 kb antisense intronic lncRNA located in the DSCAM (Down Syndrome Cell Adhesion Molecule) gene. DSCAM-AS1 was firstly described as an estrogen-responsive expressed sequence tag in an attempt to find differentially expressed transcripts between benign and malignant breast tumor cells [11]. Further pioneering studies in the context of breast cancer reported DSCAM-AS1 as the most abundant estrogen receptor α (ERα)-induced lncRNA in MCF-7 breast cancer cells and to be the main distinguishing feature of the luminal subtype of breast cancer [12,13]. DSCAM-AS1, being overexpressed in breast cancer, was identified as part of a lncRNA cluster associated with worse overall survival of breast cancer patients [14]. This cluster of oncogenic lncRNAs was found to regulate TGF-beta and Jak-STAT signaling pathways [15]. DSCAM-AS1 was shown to regulate G1/S cell cycle transition and to be an independent prognostic factor of poor survival in luminal breast cancer patients treated with endocrine therapy [16]. Mechanistically, the oncogenic action of DSCAM-AS1 was demonstrated to interact with nuclear ribonucleoprotein hnRNPL in breast cancer cells, which has been found to facilitate progression of breast cancer and induce resistance to tamoxifen [14]. Furthermore, in breast cancer cells, transcription of DSCAM-AS1 was shown to be mainly activated by FOXA1 and was in turn able to affect expression of its regulators ERα and FOXA1 via interaction with YBX1, forming a positive feedback loop leading to breast cancer progression [17]. In breast cancer cell lines, DSCAM-AS1 activated proliferation and invasion via inhibition of miR-204-5p and subsequent up-regulation of *RRM2* [18].

Further studies demonstrated overexpression of DSCAM-AS1 in various other cancer entities, always being associated with poor survival, like in non-small cell lung cancer, colorectal cancer, osteosarcoma, hepatocellular carcinoma and melanoma [19,20,21,22,23].

Animal studies corroborated the oncogenic function of DSCAM-AS1, demonstrating that knockdown or deletion of this lncRNA led to reduction of tumor size of lung, breast and colorectal cancer xenografts (reviewed in [24]). The function of lncRNA DSCAM-AS1 is known to be mediated by sponging various miRNAs, thereby affecting the mRNA landscape [24].

During preparation of this manuscript, in the end of 2021, a first study on the role of DSCAM-AS1 in endometrial cancer was published, which reported its upregulation in EC and its ability to downregulate the tumor-suppressive miR-136-5p [25]. However, the limitation of this well performed study, as stated by the authors, was that the detailed mechanisms of DSCAM AS1 in EC were not fully investigated. Thus, we think the results presented in this study add significant data to further elucidate the role of this lncRNA in endometrial cancer.

In the present study, we examined the role of DSCAM-AS1 in endometrial cancer, employing both in silico and in vitro approaches, focusing on identification of genes and pathways regulated by this lncRNA using transcriptome and pathway analyses. First, we compared DSCAM-AS1 expression in normal endometrium and endometrial cancer (EC) tissue using open-source data and searched for genes correlated with this lncRNA in EC. In the in vitro part of this study, we knocked down its expression in RL95-2 and HEC-1B EC cell lines by means of RNAi and analyzed growth and transcriptome alterations (using Affymetrix DNA Microarrays) triggered by this knockdown. Finally, we tested whether the genes being regulated after DSCAM-AS1 knockdown would be correlated with this lncRNA in endometrial cancer tissue.

## 2. Materials and Methods

### 2.1. Materials

OptiMEM medium was purchased at Invitrogen (Karlsruhe, Germany). DMEM/F12 culture medium, FBS, sodium pyruvate, insulin, L-glutamine and Accutase were obtained from Sigma-Aldrich (Munich, Germany). DSCAM-AS1 siRNAs were from ThermoFisher (Woodward, PA, USA). Affinity Script Multi Temperature cDNA Synthesis Kit was from Agilent (Santa Clara, CA, USA). RNeasy Mini Kit, RNase Free DNase Set and Quantitect SYBR Green PCR Kit were bought from Qiagen (Hilden, Germany). PCR primers were synthesized at Eurofins Genomics (Ebersberg, Germany). Transfectin reagent was obtained from BioRad (Hercules, CA, USA).

### 2.2. RNA Isolation and RT-qPCR

Total RNA was extracted from endometrial cancer cell lines by means of the RNeasy Micro Kit (Qiagen) according to the manufacturer’s protocol. After that, RNA concentration and purity was determined using a spectrophotometer. Next, 300 ng RNA from each sample was reversely transcribed to cDNA by means of the Affinity Script Multi Temperature cDNA Synthesis Kit according to the manufacturer’s protocol, using 80 ng/µL random hexamer primers (Invitrogen, Karlsruhe, Germany) and 10 mM dNTP mixture (Fermentas, St. Leon-Rot, Germany). After reverse transcription, the levels of the resulting cDNAs representing DSCAM-AS1 lncRNA and other RNAs of interest were determined by qPCR using β-actin as reference. From each sample, 4 µL cDNA were examined using the LightCycler^®^ FastStart DNA MasterPLUS SYBR Green I reagent (Roche Diagnostics GmbH, Mannheim, Germany) and 5 mM of each primer (Table S1). PCR primers (synthesized at Eurofins, Germany) were designed intron spanning to avoid amplification of genomic contaminations. qPCRs were carried out in a LightCycler^®^ 2.0 Instrument (Roche, Mannheim, Germany) under the following conditions: initial denaturation at 95 °C for 15 min, followed by 35–40 cycles containing 10 s denaturation at 95 °C, 5 s annealing at 60 °C (primer annealing temperature) and 12 s extension at 72 °C. A standard melting curve analysis finished the PCR program. Negative controls were prepared by adding distilled water instead of cDNA. To verify the identity of the PCR products, their size was first examined in 1.5% agarose gels stained with ethidium bromide. After the size check, at the first experiment, each PCR product was eluted and purified using the “QIAquick Gel Extraction Kit” (Qiagen, Hilden, Germany), and its identity finally was verified by sequencing (Eurofins MWG Operon, Ebersberg, Germany). RT-qPCR data were analyzed using the comparative ΔΔCT method calculating the difference between the threshold cycle (Cp) values of the target and reference gene of each sample and, if necessary, comparing these ΔCp values between different samples [26,27].

### 2.3. Western Blot Analysis

For preparation of cell lysate, cells were lysed in RIPA buffer (1% (*v*/*v*) Igepal CA-630, 0.5% (*w*/*v*) sodium deoxycholate and 0.1% (*w*/*v*) sodium dodecyl sulphate (SDS) in phosphate-buffered solution (PBS) containing aprotinin and sodium orthovanadate. Aliquots of cell lysate containing 10 µg of protein were resolved by 10% (*w*/*v*) SDS–polyacrylamide gel electrophoresis, followed by electrotransfer to a PVDF hybond (Amersham, UK) membrane. Immunodetection was carried out using antibodies anti-PARP (#9542 Cell Signaling; 1:1000), anti-cleaved Caspase-3 (#9661 Cell Signaling; 1:1000), anti-TRAIL (#3219 Cell Signaling; 1:2000), anti-DR4/TRAIL-R1 (#42533 Cell Signaling; 1:1000), anti-DR5/TRAIL-R2 (#8074 Cell Signaling; 1:2000), anti-Actin-HRP (HRP-60008 Proteintech; 1:30,000), anti-GAPDH-HRP (HRP-60004 Proteintech; 1:30,000) and anti-rabbit-HRP (111-035-144 Jackson Immuno Research; 1:10,000), which were detected using a chemiluminescence (ECL) system (Amersham, Buckinghamshire, UK).

### 2.4. Cell Culture, siRNA Transfection and Proliferation Studies

RL95-2 and HEC-1B endometrial adenocarcinoma cells were obtained from American Type Culture Collection (Manassas, VA, USA). Mycoplasma testing was performed on the cell lines used, and both lines have been authenticated by comparing their phenotypes microscopically with the respective images on the ATCC website. Furthermore, their different ERα status was confirmed by means of RT-qPCR. None of the cell lines, previously, has been misidentified or cross-contaminated with regard to the International Cell Line Authentication Committee. HEC-1B and RL95-2 cells were maintained in DMEM-F12 medium supplemented with 10% FCS and were cultured with 5% CO_2_ at 37 °C in a humidified incubator. For transfection with DSCAM-AS1 siRNA, 4 × 10^5^ cells per well of a 6-well dish were seeded in DMEM/F12 containing 10% FCS. The next day, cells were treated with 60 nM siRNA in OptiMEM reduced serum medium using eight µL of Transfectin reagent (BioRad, Hercules, CA, USA). For knockdown (KD) of DSCAM-AS1 expression, we used an equimolar mixture of three different pre-designed Silencer siRNAs (20 nM each) (n498114, n498116 and n508114, ThermoFisher, Waltham, MA, USA), targeting different regions of DSCAM-AS1 lncRNA. As a negative control siRNA verified not to interact with any human RNA; we used 60 nM of the Silencer Negative Control #1 siRNA (AM4611, ThermoFisher). Three and four days after siRNA treatment, cells were harvested, and total RNA was isolated as described above.

Parallel to RT-qPCR experiments testing the efficacy of siRNA-triggered lncRNA knockdown, the transfected cells, each 100 µL per well containing 2000 cells, were seeded in triplicates in a 96-well chamber in DMEM-F12 containing 10% FCS. On days 0, 3, 4, 5 and 6 after transfection, relative numbers of viable cells were measured in comparison to cells treated with negative control siRNA using the resazurin-based Cell Titer Blue (CTB) assay (Promega, Madison, WI, USA) according to the manufacturer’s instructions, and viable cell numbers were determined at 560 Ex/590 Em nm in a Victor3 multilabel counter (PerkinElmer, Waltham, MA, USA). Cell growth was expressed either as percentage of day 0 or as percentage of the solvent controls.

### 2.5. Apoptosis Assays

We used different experimental approaches to examine activation of apoptosis triggered by DSCAM-A1 knockdown. First, we used Western blot analysis for detection of cleaved PARP1 and cleaved caspase 3; further methodological and antibody information is indicated in chapter 2.4. Secondly, we employed the Caspase-Glo 3/7 assay (Promega, Madison, WI, USA), a luminometric method for detection of phosphorylation of caspases 3 and 7, according to the manufacturer’s protocol. As a positive control, cells were treated with apoptosis inducer staurosporine (1 µM). Luminescence was measured in a Victor3 multilabel counter.

### 2.6. Transcriptome Analyses Using Clariom S Human Microarrays

For hybridization of the employed Affymetrix Human Clariom S microarrays (Thermo Fisher Scientific, Munich, Germany), two biological replicas of RNA from both cell lines were isolated 72 h after siRNA transfection by means of the RNeasy Micro Kit (Qiagen) according to the manufacturer’s protocol. Sample preparation for microarray hybridization was carried out as described in the Affymetrix GeneChip^®^ Whole Transcript (WT) Sense Target Labelling Assay manual (Affymetrix, Inc., Santa Clara, CA, USA). In brief, 300 ng of total RNA were used to generate double-stranded cDNA. First, cRNA was synthesized (WT cDNA Synthesis and Amplification Kit, Affymetrix), purified and reverse transcribed into single-stranded (ss) DNA. Purified ssDNA was then fragmented and labelled with biotin (WT Terminal Labelling Kit, Affymetrix). Finally, 2.3 µg DNA were hybridized to Human Clariom S microarrays (Affymetrix) for 16 h at 45 °C in a rotating chamber. Hybridized arrays were washed and stained in the Affymetrix Washing Station FS450 using Hyb, Wash & Stain Kit (Affymetrix), and the fluorescent signals were measured in the Affymetrix GeneChip^®^ Scanner 3000-7G. For data analysis, by means of the RMA algorithm in the Affymetrix GeneChip Expression Console Software, summarized probe signals were created. They were exported to Microsoft Excel, and average signal values and comparison fold changes were calculated. Probe sets with a fold change above 2.5-fold and a student’s *t* test *p* value lower than 0.05 were considered to be regulated. Microarray processing and measurement were performed at the Affymetrix Service Provider and Core Facility, “KFB—Center of Excellence for Fluorescent Bioanalytics” (Regensburg, Germany; http://www.kfb-regensburg.de).

### 2.7. In Silico Analyses

For this study, we had to use different open-source datasets and online platforms, depending on data availability and suitability of the provided analysis tools. Data on DSCAM-AS1 expression in endometrial cancer or normal endometrium were not existing in the datasets accessible at all platforms since it is a relatively recently identified gene. Furthermore, a considerable amount of data on DSCAM-AS1 could not be found from all cancer entities; indeed, for EC, often only data from small sample numbers were available. Thus, we decided to use platforms providing the highest sample numbers for each specific analysis. For survival analysis, we used the Kaplan-Meier Plotter platform in the pan-cancer section providing RNA-seq data (including DSCAM-AS1) from 543 EC patients [28]. For correlation analyses of two candidate genes, we judged the GEPIA2 platform [29] to be the best choice, since it provided a wide range of analysis tools, the option to normalize gene expression data using a housekeeping gene and a TCGA/GTEx dataset (including DSCAM-AS1) of 172 endometrial cancer samples and 91 datasets from normal endometrium. To compare DSCAM-AS1 expression in EC and normal endometrium and to identify genes correlated with DSCAM-AS1 in EC, we preferred the well-designed tools at the “R2: Genomics Analysis and Visualization Platform” (http://r2.amc.nl) and chose the largest GEO datasets containing DSCAM-AS1 data, GSE2109 (endometrial cancer, *n* = 209) and GSE51981 (normal endometrium, *n* = 71). For further analysis of the identified DEGs upon DSCAM-AS1 knockdown or genes being associated with DSCAM-AS1 expression in endometrial tissues, we employed the gene set enrichment platform GSEA with Molecular Signatures Database (MSigDB) v7.5.1 (http://www.gsea-msigdb.org/gsea/index.jsp), since this established tool retrieves functional annotation data by gene set overlap from different sources like KEGG, Reactome, GO or MSigDB Hallmark gene sets [30]. For analysis, we chose the gene family identification tool, the biological network repository NDEx and, for identification of overlapping gene sets, the GSEA MSigDB gene set collections H and C2 to C6.

### 2.8. Statistical Analysis

For statistical analysis to compare two groups, Student’s *t*-test was used. For multiple comparisons analyses, the nonparametric Kruskal-Wallis test with Dunn’s post-test was applied. Spearman’s rank test was used for correlation analyses. Statistics were performed using Prism software version 7.04 (Graph Pad, San Diego, CA, USA).

## 3. Results

### 3.1. DSCAM-AS1 Overexpression in Endometrial Adenocarcinoma

**Figure 1:**
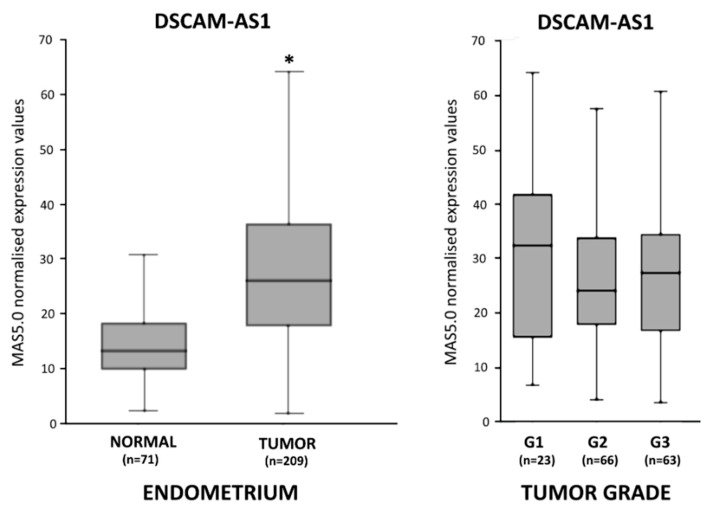
Expression of lncRNA DSCAM-AS1 in endometrial tissues as assessed by analysis of GEO open-source data using the online platform http://r2.amc.nl. Left panel: expression in normal endometrium (*n* = 71) (GEO ID: GSE51981) and endometrial adenocarcinoma (*n* = 209) (GEO ID: GSE2109). DSCAM-AS1 levels are shown as normalized expression values using the MAS5.0 method (Affymetrix). Right panel: expression in endometrial cancer grading subgroups G1, G2 and G3 (GEO ID: GSE2109). Shown graphs are box and whisker charts. Boxes represent the upper (Q3) and the lower quartile (Q1), and the line indicates the median of lncRNA expression values. The ends of the whisker are set as 1.5 × IQR above Q3 or below Q1. * = *p* < 0.01.

In our initial in silico analyses, by means of the “R2: Genomics Analysis and Visualization Platform” (http://r2.amc.nl), we first compared DSCAM-AS1 expression in normal endometrium using open-source data (GEO ID: GSE51981) and endometrial cancer tissue (GEO ID: GSE2109), both generated with GeneChip™ Human Genome U133 plus 2.0 Arrays (Affymetrix). DSCAM-AS1 RNA levels were observed to be about 2-fold higher in endometrial adenocarcinoma than in normal endometrium (*p* < 0.01) (Figure 1). When we performed a subset analysis of endometrial cancer tissue with regard to tumor grade (G1, G2 and G3), no significant difference between the subgroups was present (Figure 1, right panel).

### 3.2. Genes Correlated with DSCAM-AS1 in Endometrial Adenocarcinoma

DSCAM-AS1 expression is known to be activated by ERα signaling in breast cancer, so we now tested whether correlations of this lncRNA with ERα or its targets genes could be identified in endometrial cancer tissue. For this purpose, we analyzed open source TCGA/GTEx data of 172 endometrial cancer tissues available on the Gepia2 platform, which allowed normalization by a housekeeping gene (http://gepia2.cancer-pku.cn/) [29]. DSCAM-AS1 levels were found to be considerably associated [31] with *ESR1* expression (rho = 0.40, *p* = 8.9 × 10^−8^) and with ERα target gene *TFF1* (*PS2*) (rho = 0.41, *p* = 2.6 × 10^−8^) in endometrial adenocarcinoma tissues. Correlation with *TFF1* in normal endometrium was even stronger (rho = 0.86, *p* = 0.00019) due to the alteration of ERα levels during the menstrual cycle, which leads to equidirectional regulation of DSCAM-AS1 and *TFF1*. Progesterone receptor (*PGR*) gene as another classical ERα target was also found to be significantly associated with DSCAM-AS1 expression in endometrial cancer tissue (rho = 0.46, *p* = 4.0 × 10^−10^) but not in normal endometrium or uterine tissues (Figure S1).

We observed overexpression of DSCAM-AS1 in endometrial cancer tissue, so we now examined to what extent this lncRNA would be associated with expression of proliferation regulators and other genes involved in carcinogenesis. For this purpose, we used the correlation tool at “R2: Genomics Analysis and Visualization Platform” (http://r2.amc.nl) to search for genes correlated with DSCAM-AS1 in a subset of 137 endometrioid endometrial adenocarcinomas (GSE2109). Setting the R correlation coefficient to a cut-off value of 0.4, to include only genes with moderate to strong correlation (applying Bonferroni multiple testing correction), DSCAM-AS1 was positively correlated with 761 genes and negatively correlated with 148 genes (Supplemental File S1).

First, the gene family analysis tool at GSEA website (http://www.gsea-msigdb.org/) [30] notably identified prolactin (*PRL*) gene was positively correlated with DSCAM-AS1, coding for a hormone being a highly potent driver of endometrial cancer development and progression (R = 0.497, *p* = 2.41 × 10^−8^) (reviewed in [32]). Corroborating the significance of this correlation, DSCAM-AS1 was also associated with expression of *PRLH* gene coding for the prolactin releasing hormone (R = 0.503, *p* = 1.53 × 10^−8^). Association of DSCAM-AS1 with both *PRL* and *PRLH* is an important observation which should be considered in the attempt to assess the role of this lncRNA in EC. Among the genes most significantly associated with DSCAM-AS1 were *EPOR* and *CYP1A2*, both clearly associated with short OS in EC [28,33]. Among the 148 genes negatively correlated with DSCAM-AS1 in endometrioid endometrial adenocarcinoma, the gene family tool identified tumor suppressor *SDHB*, differentiation marker *CD9* and TFs *CAND1, GTF2A2 PLRG1, TAF10, YBX1* and *ZNF532.*

Next, using GSEA, we searched for overlaps of the genes positively correlated with DSCAM-AS1 in endometrial cancer with the gene sets H, GO and C2 to C6, first resulting in identification of 31 genes, including *CYP1A2*, all being high confidence targets of the miRNAs miR-373-5p, miR-371B-5p and miR-616-5p. No interaction of DSCAM-AS1 with these miRNAs is known, so it can only be speculated that this lncRNA might suppress the function of these miRNAs, which would explain the data. To our surprise, GSEA did reveal only a few further gene set overlaps. Among the curated gene sets, there was an overlap (12 of 63 genes) with gene set “Liu common cancer genes” containing “Low abundance transcripts common to nasopharyngeal carcinoma (NPC), breast and liver tumors”. With regard to canonical pathways using all corresponding databases, only a weakly significant overlap (5 of 30 genes) with REACTOME TRAF6 MEDIATED IRF7 ACTIVATION [30] was identified (Table 1a).

GSEA analysis of the 148 genes negatively correlated with DSCAM-AS1 in EC, among others, revealed an overlap with a REACTOME gene set involved in regulation of stability and activity of the important EC tumor suppressor PTEN (Table 1b).

### 3.3. Knockdown of DSCAM-AS1 in Endometrial Cancer Cell Lines

**Figure 2:**
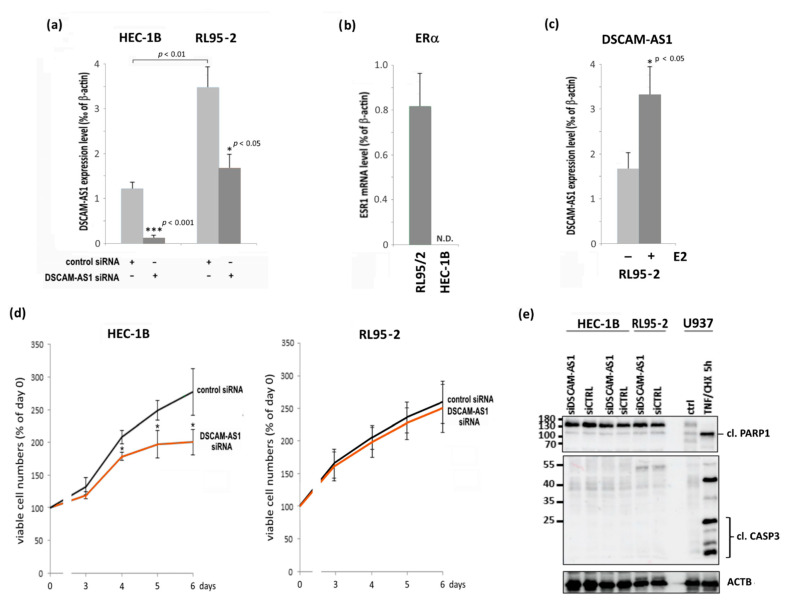
Effects of a knockdown of DSCAM-AS1 expression by means of siRNA transfection. (**a**) HEC-1B and RL95-2 endometrial adenocarcinoma cells were transfected with 60 nM negative control siRNA or the same amounts of siRNAs specific for DSCAM-AS1. A total of 96h after transfection, total RNA was isolated, and cDNA was subjected to RT-qPCR analysis as described in the methods section. DSCAM-AS1 expression levels were normalized to ACTB expression (ΔCT). * *p* < 0.05, *** *p* < 0.001 vs. negative control siRNA (*n* = 3). (**b**) RT-qPCR analysis of ERα (ESR1) expression in the indicated cell lines. ESR1 transcript levels were normalized to ACTB expression. Knockdown was less efficient in RL95-2 cells due to its positive ERα status (see Discussion section). N.D. = not detectable (*n* = 3). (**c**) Effect of E2 (17β-estradiol, 3 nM) on DSCAM-AS1 expression in RL95-2 cells. Cells were treated with vehicle or E2 for 48 h and the isolated RNA was subjected to RT-qPCR analysis. * *p* < 0.01 vs. vehicle. (**d**) Growth of the indicated endometrial cancer cell lines after transfection with DSCAM-AS1-specific siRNA. Cells were transfected with 60 nM of negative control siRNA or DSCAM-AS1 siRNA, and relative numbers of viable cells were measured 3, 4, 5 and 6 days after transfection by means of the Cell Titer Blue assay (Promega) as described in the methods section. * *p* < 0.05 vs. control siRNA (*n* = 4). (**e**) Western blot (WB) analysis for detection of PARP1- and caspase 3-cleavage (cl.) for determination of cellular apoptosis. As a positive control, U937 cells were treated with a combination of TNFα and cycloheximide (CHX). WB analysis was performed as described in the methods section.

To elucidate the function of DSCAM-AS1 in both cell lines, we knocked down its expression by RNAi. For the in vitro part of this study, we employed the ERα-negative cell line HEC-1B and the ERα-positive cell line RL95-2. We first verified the ERα status of both cell lines using RT-qPCR and demonstrated that mRNA of this receptor was present in RL95-2 cells only (Figure 2b). Further analysis revealed DSCAM-AS1 levels to be 2.8-fold higher in RL95-2 than in HEC-1B cells lacking ERα (*p* < 0.01) (Figure 2a). Transfection of ERα-negative HEC-1B cells with DSCAM-AS1 siRNA efficiently reduced the levels of this lncRNA down to 9.5% (*p* < 0.001) 96 h after transfection when compared to cells treated with negative control siRNA. In contrast, the same siRNAs exhibited a notably smaller effect on the ERα-positive line RL95-2, decreasing DSCAM-AS1 levels by only 51.9% (*p* < 0.05) (Figure 2a). A similar efficient knockdown in HEC-1B cells was detected 48h and 72h post transfection, but its presence after 96h was important for interpretation of the following growth assay. To verify presence of the reported ERα-dependent activation of DSCAM-AS1 expression, we treated the ERα-positive endometrial cancer line RL95-2 with 17β-estradiol (E2). After 48 h of treatment, RT-qPCR analysis of the isolated RNA showed a significant increase of DSCAM-AS1 levels (Figure 2c).

Now we tested to what extent knockdown of this lncRNA would affect viable cell numbers of both lines. In HEC-1B cells, the robust DSCAM-AS1 knockdown resulted in a significant decline of viable cell numbers 4, 5 and 6 days after transfection, with a maximum inhibition by 54.3% on day 6 (*p* < 0.01 vs. negative control siRNA) (Figure 2d). In contrast, the weakly pronounced knockdown in ERα-positive RL95-2 cells did not significantly affect growth of this cell line. Negative control RNA did not affect DSCAM-AS1 expression or cell growth when compared to cells transfected without siRNA (data not shown). We now were eager to find out whether the KD would activate apoptosis, which could contribute to the decrease of viable cell numbers observed after DSCAM-AS1 knockdown in HEC-1B cells. However, neither the Western blot analysis of cleaved PARP1 nor of cleaved caspase 3 showed any cleavage product being present in the positive control, which was U937 cells treated with TNFα and cycloheximide (Figure 2e). Examination of caspase 3/7 activation (Caspase-Glo 3/7 assay, Promega) did not show any change of basal activity of these caspases after knockdown of DSCAM-AS1 (data not shown).

### 3.4. Transcriptome Alterations after DSCAM-AS1 Knockdown in Endometrial Cancer Cells

**Figure 3:**
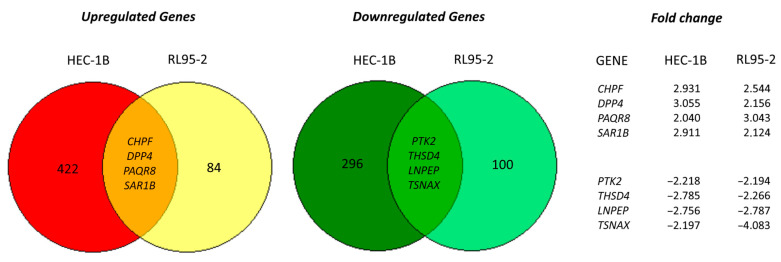
Venn diagram of the genes up- or downregulated upon DSCAM-AS1 silencing in HEC-1B and RL95-2 cells, with the overlapping regions indicating genes with equidirectional regulation in both cell lines. (Cut-off value: 2-fold change vs. control siRNA).

As we observed both DSCAM-AS1 to be overexpressed in endometrial cancer tissue and growth inhibition of HEC-1B cells after its knockdown, we now examined transcriptome alterations and signaling pathways which might underlie these observations. By means of DNA microarray analysis (Affymetrix), we examined transcriptome changes triggered by knockdown of DSCAM-AS1 in HEC-1B and RL95-2 cells. DSCAM-AS1 knockdown in HEC-1B cells resulted in at least 2-fold downregulation of 300 genes (including 25 ERα inducible genes and cell differentiation genes like PSG1 or ITGB3) and upregulation of 426 genes (including SUFU tumor suppressor) 96 h after transfection (analyzed by GSEA (http://www.gsea-msigdb.org/)). In RL95-2 cells, the less-pronounced knockdown of this lncRNA led to downregulation of 104 genes and upregulation of 88 genes using the same cut-off value. Venn diagram analysis revealed four genes to be downregulated in both cell lines and another four genes to be upregulated in HEC-1B as well as RL-95/2 cells (Figure 3). Although the knockdown in RL95-2 cells was unsatisfactory, when we examined the clinical relevance of these eight genes in endometrial cancer in terms of association with patients’ survival using the RNA-seq data from 543 EC patients [28] provided by the database of the Kaplan-Meier Plotter website https://kmplot.com, we observed that the upregulated genes CHPF, PAQR8 and SAR1B were associated with longer overall survival (OS), whereas the downregulated genes PTK2, THSD4 and LNPEP were significantly associated with a shorter OS (PTK2) or showed a trend towards adverse OS in this cancer entity (Figure S2).

Genes exhibiting the highest regulation after DSCAM-AS1 knockdown are shown in Table 2. To further characterize these genes with regard to their function in cancer, we examined their clinical significance by testing their association with overall survival (OS) in endometrial cancer as described above (https://kmplot.com, *n* = 543 EC patients) [28]. Notably, four genes with the strongest downregulation in HEC-1B cells (*SCEL, TMC7, ELL2* and *UNC13D*) were significantly associated with a shorter OS of EC patients (Figure S3). With regard to the top-induced genes, *THG1L* was significantly associated with a longer OS, whereas the upregulated *STEAP2* exhibited only a trend towards a longer OS, and WNT7 was associated with a prolonged RFS but a short OS. The data from RL95-2 microarray analysis are shown but should not be overinterpreted due to the insufficient knockdown addressed before.

**Figure 4:**
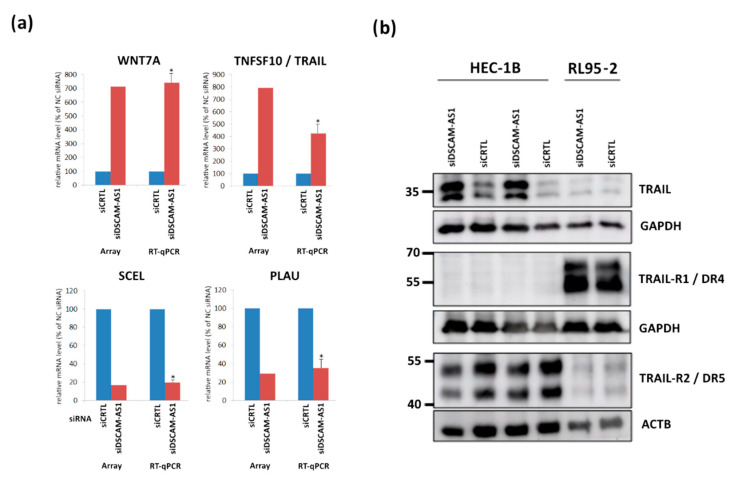
(**a**) Validation of the microarray results by analyzing expression of the indicated genes after DSCAM-AS1 knockdown by means of RT-qPCR comparing the results of both methods. Total RNA from cells transfected with negative control siRNA (siCTRL, NC) (AM4611, Thermo Fisher) was used as internal control. (**b**) Western blot analysis of TNFSF10 gene product TRAIL, significantly upregulated on the mRNA level in HEC-1B cells, and of TRAIL receptors DR4 and DR5. Shown are exemplary WB results. As loading controls, housekeeping genes GAPDH and ACTB were analyzed. * *p* < 0.01 vs. siCTRL.

We then exemplarily confirmed the microarray data by means of RT-qPCR analysis of DSCAM-AS1 KD-triggered regulation of the genes TNFSF10 (coding for TRAIL), PLAU, WNT7A and SCEL. Examination on the mRNA level verified the microarray results of all four genes (Figure 4a). PCR primer sequences can be found in supplemental Table S1. Western blot analysis confirmed the notable increase of TNFSF10 gene expression by detection of elevated TRAIL protein levels in HEC-1B cells (Figure 4b). Additionally, we examined expression of TRAIL receptors DR4 and DR5, observing that DR5, but not DR4, was expressed on the protein level in HEC-1B cells, suggesting TRAIL sensitivity to be impaired in this cell line.

We now annotated all at least 2.5-fold regulated genes in DSCAM-AS1 siRNA-transfected HEC-1B cells to the Gene Ontology (GO) category “Biological processes” using the software GOTermFinder (https://go.princeton.edu/cgi-bin/GOTermFinder). From the results, gene ontology terms were selected that had a significant corrected *p*-value and additionally indicated the direction of regulation of the biological process, which was helpful to interpret the results. Analysis of the down-regulated genes notably suggested activation of tumor-promoting biological processes by DSCAM-AS1 (Table 3). Among the downregulated genes were several known to activate proliferation of tumor cells (NOTCH1, HMGA2, PTK2, FOSL1, GREM1 and EGR1). In contrast, GO-term annotation of the upregulated genes suggested DSCAM-AS1 inhibits cellular differentiation and tumor-suppressing biological processes (Table 3).

Next, for a more detailed characterization of the DEGs regulated upon DSCAM-AS1 silencing in HEC-1B cells, we used the GSEA gene set enrichment platform (http://www.gsea-msigdb.org/gsea/index.jsp) and the (v7.5.1 MSigDB) database [30]. First, gene family analysis of the genes downregulated upon DSCAM-AS1 knockdown identified three oncogenes, *HMGA2, LHFPL6* and *NOTCH1*, and an integrated NDEx query [34] identified six genes of the pathway “Activating invasion and metastasis”, namely *NOTCH1, CAPN2, CLDN4, COL3A1, F2RL2* and *MAP2K3*, further supporting the oncogenic role of DSCAM-AS1. Computing overlaps with the MSigDB database (gene set collections H and C2 to C6) resulted in identification of several highly significant gene set overlaps, including tumor-promoting gene sets of breast cancer cells or cholangiocarcinoma (Table S2). Analyzing the genes upregulated after DSCAM-AS1 knockdown on the same platform, gene family analysis first identified an upregulated tumor suppressor, *SUFU*, and eleven differentiation markers (*PSG1, ALCAM, C5AR1, CSF3R, DPP4, IL10RB, IL6ST, ITGB3, NECTIN1, SEMA4D* and *TNFSF10*), suggesting this oncogenic lncRNA to suppress differentiation. Analysis of overlaps of the upregulated genes with defined gene sets showed, among others, a highly significant overlap with transcripts being high confidence targets of miR-217-5p, a miRNA so far not being identified as a target of DSCAM-AS1, but which has been demonstrated to act as a tumor-suppressor miRNA, inhibiting proliferation and invasion in vitro and being downregulated in tumor tissue of various cancer entities [35,36,37,38,39,40]. This analysis identified further significant overlaps with gene sets containing high confidence target mRNAs of miR-8485, miR-6807-3p, miR-548AV-5P/miR-548K and miR-8054, the role of which in cancer has been little studied. The genes induced upon DSCAM-AS1 silencing also showed significant overlaps with sets of genes induced in late stages of differentiation of embryoid bodies from embryonic stem cells and with a set of genes coordinately upregulated in a compendium of adult tissue stem cells (Table S2).

**Figure 5:**
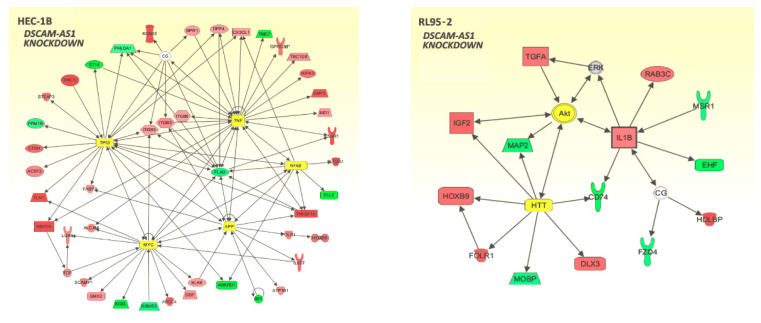
Networks of genes regulated upon silencing of DSCAM-AS1 in HEC-1B and RL95-2 cells as assessed by Affymetrix GeneChip and subsequent pathway analyses using Ingenuity Pathway Analysis (IPA) software (Qiagen Bioinformatics). Knockdown was less efficient in RL95-2 cells due to its positive ERα status (see Discussion section). The gene networks of upregulated genes (red) and downregulated genes (green) also indicate their key upstream regulators (yellow). Solid arrows: affecting gene expression.

By means of IPA Pathway Analysis software (Qiagen Bioinformatics), we then identified networks connecting genes regulated after DSCAM-AS1 knockdown and their key upstream regulators (Figure 5). Knockdown of this lncRNA by at least 90% (in HEC-1B cells) led to regulation of gene networks controlled by the upstream regulators tumor necrosis factor (TNF), tumor protein 53 (TP53) and by transcription factors MYC and NFκB. The transcriptome of RL95-2 cells was less affected due to insufficient DSCAM-AS1 knockdown resulting from ERα-triggered DSCAM-AS1 induction. Thus, with regard to RL-95-2 cells, the results of Ingenuity pathway analysis (IPA) software (Qiagen) should be assessed with caution. This software suggested AKT Serine/Threonine Kinase 1 to be a central molecule of this network. As important upstream regulators, both the huntingtin (HTT) gene and the upregulated cytokine interleukin 1β (IL1B) were identified, both leading to up- or downregulation of their target genes.

### 3.5. Correlation of DSCAM-AS1 with Knockdown DEGs in Endometrial Adenocarcinoma Tissues

Next, we tested whether the link between DSCAM-AS1 and the genes regulated upon its knockdown in vitro, as suggested from the transcriptome alterations of endometrial cancer cell lines (particularly HEC-1B), would also be observable in endometrial cancer tissue. For this purpose, we searched for correlations between expression of DSCAM-AS1 and these DEGs present in EC tissues. Using the GEPIA2 online tool and gene expression data from 170 endometrial adenocarcinomas, Spearman rank correlation revealed a positive association of DSCAM-AS1 with the 4.7-fold downregulated tumor-promoting gene ELL2 (rho = 0.42, *p* = 3.5 × 10^−9^) and with the downregulated tumor-promoting genes UNC13D (FHL3, Munc13-4) (rho = 0.39, *p* = 3 × 10^−8^) and MAP2 (rho = 0.4, *p* = 1.7 × 10^−11^) in endometrial cancer tissues (Figure S4). In contrast, we observed a negative correlation of DSCAM-AS1 with the highly upregulated WNT7A gene (rho = −0.25, *p* = 5.3 × 10^−5^) (HEC-1B), which is reported to act as tumor suppressor in endometrium [41]. These correlation data clearly corroborated the hypothesis that DSCAM-AS1 is involved in the regulation of important cancer-related genes in this cancer entity.

### 3.6. Expression of DSCAM-AS1 and Survival of Endometrial Cancer Patients

**Figure 6:**
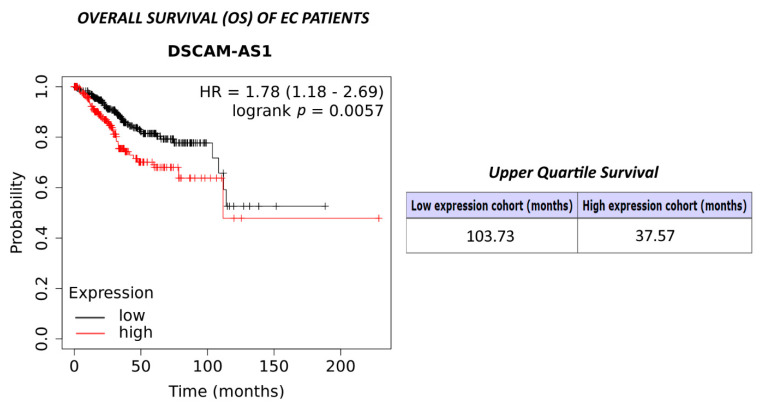
Kaplan-Meier plot showing the overall survival (OS) of 543 women with endometrial adenocarcinoma (EC) with high or low levels of DSCAM-AS1. The analysis was carried out using the data and software available at http://kmplot.com/analysis/index [28].

Using the data and software at http://kmplot.com/analysis/index, in the module with pan-cancer RNAseq and survival data, 543 patients with endometrial adenocarcinoma were included [28]. Kaplan-Meier analysis revealed that women with high intratumoral expression of DSCAM-AS1 had a significantly shorter overall survival (OS) than patients with lower expression of this lncRNA (HR = 1.78 (1.18–2.69), log rank *p* = 0.0057) (Figure 6). The lower quartile OS of the high expression cohort was 37.57 months, whereas the upper quartile OS of the low expression cohort was 103.73 months. With regard to relapse-free survival (RFS), no significant difference between both groups was observed.

## 4. Discussion

An increasing number of studies have shown that lncRNAs play important roles in cancer. With regard to endometrial cancer, in the recent years a number of studies have examined the function of various lncRNAs in this cancer entity [6,8,10,25,42,43,44,45,46]. Overlapping with our ongoing study, in the end of 2021 a study has been published reporting DSCAM-AS1 overexpression in endometrial cancer tissue and suggested this lncRNA facilitates cancer progression by upregulation of miR-136-5p [25]. However, as the authors state, a limitation of their well-performed study was not having investigated the detailed mechanisms of DSCAM-AS1 in EC. In this regard, the results of our study add important data further elucidating the mechanisms underlying the functions of this lncRNA by identification of genes and pathways regulated by DSCAM-AS1 in endometrial cancer cells, being corroborated by demonstrating correlation between several genes found to be regulated after its knockdown with DSCAM-AS1 in endometrial cancer tissues.

Our analyses demonstrating elevated expression of DSCAM-AS1 in endometrial tumor tissue are in line with studies reporting increased DSCAM-AS1 levels in several cancer types [11,12,13,17,24]. In non-small cell lung cancer, high expression of DSCAM-AS1 was associated with shorter overall survival and was suggested to act as an oncogene [12]. With regard to breast cancer, different studies report this lncRNA to be overexpressed, to promote tumor growth and to be an independent prognostic factor of poor survival in ductal carcinoma of the breast and in luminal breast cancer patients treated with endocrine therapy [16,18,47]. In a study to identify ERα- and breast cancer-associated lncRNAs, DSCAM-AS1 was the lncRNA being most dependent on ERα expression. It exhibited the strongest induction after estrogen stimuli, and it was able to promote tumor progression and tamoxifen resistance [14]. Expression of DSCAM-AS1 was shown to be activated both by E2-bound ERα and its unliganded form. The reported upregulation of this lncRNA by ERα, which we showed to be also present in ERα-positive RL95-2 cells, counteracting siRNA-mediated DSCAM-AS1 knockdown in this cell line, is suggested to underlie the positive correlation of DSCAM-AS1 with expression of ERα and its target genes *TFF1* (*PS2*) and *PGR* observed in our in-silico analyses.

DSCAM-AS1 in EC tissue was considerably associated with expression both of *PRL* gene coding for prolactin, a highly potent driver of endometrial cancer development and progression, and with *PRLH* gene, coding for prolactin releasing hormone, and the results of GSEA showed an overlap of the positively correlated genes with a set of common cancer genes; thus, it is tempting to speculate that these results support the oncogenic role of this lncRNA in this cancer entity. The identification of an overlap of 31 positively correlated genes with high confidence targets of miR-373-5p, miR-371B-5p and miR-616-5p might point to an interaction of DSCAM-AS1 with these miRNAs, which must be examined in further studies. GSEA of the genes negatively correlated with this lncRNA showed an overlap with genes involved in regulation of the important endometrial tumor suppressor *PTEN*; however, this result is deemphasized by the lack of any correlation between DSCAM-AS1 and *PTEN* itself.

The association of high DSCAM-AS1 levels with poor overall survival (OS) we observed after in silico analysis of open-source data of 543 endometrial cancer patients is in line with similar findings in breast cancer, melanoma, non-small cell lung cancer, colon cancer and osteosarcoma [15,18,19,21,23,47].

For the in vitro part of our study, we employed endometrial cancer cell lines with different ERα status, ERα-negative HEC-1B and the ERα-positive RL95-2 cells. Our observations that DSCAM-AS1 levels were 2.8-fold higher in RL95-2 than in HEC-1B cells are suggested to result from the reported induction of DSCAM-AS1 expression by ERα [12,13]. The different ERα status of these lines and the resulting distinct knockdown efficacy (despite similar transfection efficiencies) is also suggested to underlie the different knockdown response in terms of growth inhibition and transcriptome alterations. The absence of significant growth-inhibitory actions of the weak DSCAM-AS1 knockdown in RL95-2 cells may result, apart from the insufficient knockdown resulting from permanent ERα-induced upregulation of DSCAM-AS1, from the presence of ERα in RL95-2 cells known to enhance their proliferation via ERα activation by estrogens present in untreated FBS. In contrast, the considerable knockdown of DSCAM-AS1 in ERα-negative HEC-1B cells resulted in significant growth inhibition, being in line with the results of various in vitro studies on cell lines of different tumor entities like non-small cell lung cancer, colorectal cancer, osteosarcoma, hepatocellular carcinoma and melanoma, [20,48,49,50,51], suggesting that the oncogenic action of this lncRNA is not dependent on the presence of ERα, but is upregulated by this receptor in estrogen-responsive cancer and cell lines (endometrial, cervical and breast cancer) [14,24]. Notably, DSCAM-AS1 silencing led to downregulation of 25 ERα-target genes, suggesting that this lncRNA might affect estrogen signaling even in the absence of functional ERα.

The results of the GSEA analysis of genes upregulated upon DSCAM-AS1 knockdown, identifying 11 differentiation genes and significant overlaps with sets of genes induced in late stages of differentiation of embryoid bodies from embryonic stem cells and with a set of genes coordinately upregulated in a compendium of adult tissue stem cells, suggests that DSCAM-AS1 inhibits expression of differentiation genes, supporting its oncogenic function in EC.

Notably, among the genes downregulated upon DSCAM-AS1 knockdown were several proliferation activators of endometrial (and other) cancer cells, like *NOTCH1* [52,53], *HMGA2* [54], *PTK2/FAK* [55,56], *FOSL1* [57], *GREM1* [58] and *EGR1* [59]. Downregulation of these proliferation inducers most likely is the main molecular mechanism underlying the growth-inhibitory effect of DSCAM-AS1 silencing on HEC-1B cells.

Additionally, knockdown of DSCAM-AS1 triggered a broad anti-tumoral transcriptome response, revealed by Ingenuity pathway analysis software (IPA, Qiagen Bioinformatics), showing networks of genes regulated by DSCAM-AS1 silencing connected by NFκB, TP53 and TNFα as key regulators. Several of the genes exhibiting the strongest up- or downregulation are known to affect cancer cell growth. The tumor necrosis factor (ligand) superfamily, member 10 (*TNFSF10*) (TRAIL), upregulated 7.92-fold in HEC-1B cells with low DSCAM-AS1 levels, cells, has been reported to preferentially induce apoptosis or necroptosis in transformed and tumor cells and to decrease cancer cell growth [60]. The fact that we did not observe any sign of TRAIL-triggered apoptosis might be explained by the missing expression of death receptor 4 (DR4) in HEC-1B cells, since loss or down-regulation of DR4 is known to desensitize cells to TRAIL-triggered cell death. *WNT7a* gene (7.15-fold up-regulated) codes for a secreted ligand of the wingless (WNT) family, which not only guides the development of the anterior-posterior axis in the female reproductive tract but also plays a critical role in uterine smooth muscle pattering and maintenance of adult uterine function [61]. With regard to cancer, *WNT7a* seems to have pleiotropic functions. On the one hand, this gene is reported to promote growth of different tumor types by activation of the canonical Wnt pathway [62,63]. On the other hand, numerous studies reported *WNT7a* to exert anti-tumoral functions in different tumor entities. Loss of WNT7a protein was reported to be a major contributing factor for increased lung tumorigenesis [64]. WNT7a deficiency was shown to predict worse disease-free and overall survival in estrogen receptor-positive breast cancer [65]. *WNT7a* was also found to decrease proliferation of cervical cancer cells [66]. Importantly, a recent study suggested *WNT7a* to decrease growth of endometrial cancer cell lines [67]. Thus, *WNT7a* is also able to exert tumor-suppressive effects. Our data suggest downregulation of *WNT7a* by DSCAM-AS1, which is overexpressed in endometrial cancer, so this interaction might contribute to endometrial carcinogenesis. Sciellin (*SCEL*) gene, being downregulated about 6-fold after knockdown of DSCAM-AS1, has been recently reported to promote invasion and metastasis of colon cancer (CRC) cells, to increase WNT signaling by activating β-catenin and its downstream target c-myc, and to activate mesenchymal-to-epithelial transition (MET) through a SCEL-β-catenin-E-cadherin axis [68]. Thus, sciellin has been suggested to be a useful therapeutic target for preventing or eliminating CRC hepatic metastasis. Furthermore, sciellin was found to be as marker for papillary renal cell tumors [69]. No studies on sciellin in endometrial cancer exist, so we examined its expression by means of GEPIA2 online analysis (http://gepia.cancer-pku.cn/detail.php?gene=scel), showing a 6.5- fold increased *SCEL* expression in endometrial cancer compared to normal tissue and a decreased overall survival of endometrial cancer patients with higher *SCEL* expression (HR 2.1, *p* = 0.049) [29]. Thus, our data suggest that DSCAM-AS1 might also have a tumor-promoting role in endometrial carcinogenesis via activation of sciellin. The *PLAU* gene (also called uPA, urokinase plasminogen activator), being 3.39-fold downregulated in HEC-1B cells exhibiting lower DSCAM-AS1 levels, encodes a secreted serine protease converting plasminogen to plasmin. *PLAU* acts as tumor-promoting in most cancer entities since its expression leads to ECM degradation, invasion of tumor cells and tumor progression, and high *PLAU* expression typically is associated with an unfavorable prognosis [70]. Suppression of this gene and the related receptor has also been reported to inhibit proliferation of cancer cells of different origin in vitro via PI3K/AKT and ERK/p38 signaling pathways [71]. Thus, our data indicating *PLAU* expression is activated by DSCAM-AS1 also point to a tumor-promoting role of this lncRNA in this context.

Finally, the results of our correlation analyses of DSCAM-AS1 expression in endometrial cancer tissues with cancer-related genes regulated after its knockdown in vitro, revealing positive associations with genes downregulated and negative correlations with genes upregulated upon DSCAM-AS1 knockdown, further support that the oncogenic functions of this lncRNA as determined by in vitro transcriptome analysis and functional assays particularly on HEC-1B cells are present in endometrial cancer tissue.

## 5. Conclusions

The observed overexpression of DSCAM-AS1 in endometrial cancer, its association with tumor-promoting genes in endometrial cancer tissue and the effects of DSCAM-AS1 silencing, resulting in growth inhibition of HEC-1B cells via downregulation of proliferation activators and a broad anti-tumoral transcriptome response, substantiate an oncogenic role of DSCAM-AS1 in endometrial cancer. In silico correlation data suggested the reported interaction between ERα and DSCAM-AS1 to be present in endometrial cancer tissue, whereas our in vitro data demonstrated the oncogenic actions of this lncRNA not to depend on expression of ERα. This study encourages attempts to examine to what extent targeting DSCAM-AS1, e.g., by antisense-oligonucleotide (ASO) approaches, might be efficient in the therapy of endometrial cancer [72].

## Figures and Tables

**Table 1:** (**a**) Gene set enrichment analysis (GSEA) of genes positively correlated with DSCAM-AS1 in endometrial cancer (GSEA, MSigDB v7.5.1) [30]. FDR = false discovery rate. (**b**) Gene set enrichment analysis (GSEA) of genes negatively correlated with DSCAM-AS1 in endometrial cancer (GSEA, MSigDB v7.5.1) [30]. Shown are the 10 most significant overlaps. FDR = false discovery rate.

**(a) GSEA OF GENES POSITIVELY CORRELATED WITH DSCAM-AS1 IN ENDOMETRIAL CANCER**				
Gene Set Name (# Genes)	Description	Genes in Overlap	*p*-value	FDR
LIU COMMON CANCER GENES (63)	Low abundance transcripts common to nasopharyngeal carcinoma (NPC), breast and liver tumors	12	2.96 × 10^−12^	9.43 × 1^−9^
miR-373-5p (956)	Genes predicted to be high confidence targets of miRBase v22 hsa-miR-373-5p in miRDB v6.0	31	1.81 × 10^−6^	1.73 × 10^−3^
miR-371-5p (959)	Genes predicted to be high confidence targets of miRBase v22 hsa-miR-371-5p in miRDB v6.0	31	1.92 × 10^−6^	1.73 × 10^−3^
miR-616-5p (961)	Genes predicted to be high confidence targets of miRBase v22 hsa-miR-616-5p in miRDB v6.0	31	1.99 × 10^−6^	1.73 × 10^−3^
REACTOME TRAF6 MEDIATED IRF7 ACTIVATION (30)	TRAF6 mediated IRF7 activation	5	1.53 × 10^−5^	2.35 × 10^−3^
**(b) GSEA OF GENES NEGATIVELY CORRELATED WITH DSCAM-AS1 IN ENDOMETRIAL CANCER**				
SCGGAAGY ELK1 02 (1242)	Genes with occurrence of the motif M3 SCGGAAGY, the ELK1 transcription factor binding site V$ELK1_02 (v7.4 TRANSFAC)	36	9.7 × 10^−23^	1.09 × 10^-−9^
GOBP PROTEOLYSIS (1790)	The hydrolysis of proteins into smaller polypeptides and/or amino acids	35	9.81 × 10^−17^	2.5 × 10^−13^
GOBP MACROMOLECULE CATABOLIC PROCESS (1331)	The chemical reactions and pathways resulting in the breakdown of a macromolecule	34	9.16 × 10^−20^	7.01 × 10^−16^
GOMF RNA BINDING (1972)	Binding to an RNA molecule	31	2.34 × 10^−12^	4.06 × 10^−9^
GOBP PROTEIN CATABOLIC PROCESS (977)	The chemical reactions and pathways resulting in the breakdown of a protein	27	1.33 × 10^−16^	2.54 × 10^−13^
GOBP CELLULAR PROTEIN CATABOLIC PROCESS (819)	The chemical reactions and pathways resulting in the breakdown of a protein by individual cells.	26	1.9 × 10^−17^	7.26 × 10^−14^
HALLMARK MYC TARGETS V1 (200)	A subgroup of genes regulated by MYC - version 1 (v1).	17	9.3 × 10^−19^	4.65 × 10^−17^
REACTOME SWITCHING OF ORIGINS TO A POSTREPLICATIVE STATE (91)	Switching of origins to a post-replicative state	14	2.29 × 10^−19^	1.85 × 10^−16^
WP PROTEASOME DEGRADATION (64)	Proteasome degradation	12	7.18 × 10^−18^	4.77 × 10^−15^
REACTOME REGULATION OF PTEN STABILITY AND ACTIVITY (69)	Regulation of PTEN stability and activity	11	1.19 × 10^−15^	8.37 × 10^−14^

**Table 2:** Top 10 regulated genes after RNAi-mediated knockdown of DSCAM-AS1 in HEC-1B and RL-95-2 cells (*p* < 0.05). The knockdown efficacy differed between both cell lines due to ERα-triggered DSCAM-AS1 upregulation in RL95/2 cells. FC = fold-change.

Genes Regulated upon DSCAM-AS1 Silencing (Top 10)					
HEC-1B			RL95-2		
Gene Symbol	Gene Name	FC	Gene Symbol	Gene Name	FC
*SCEL*	sciellin	−5.99	*EHF*	ets homologous factor	−3.14
*TMC7*	transmembrane channel like 7	−4.93	*MAP2*	microtubule associated protein 2	−2.84
*ELL2*	elongation factor, RNA polymerase II, 2	−4.70	*LNPEP*	UTR3 best transcript NM_175920	−2.79
*UNC13D*	unc-13 homolog D (C. elegans)	−3.46	*KRT23*	keratin 23, type I	−2.72
*PLAU*	plasminogen activator, urokinase	−3.39	*FADS1*	fatty acid desaturase 1	−2.69
*STEAP2*	STEAP family member 2, metalloreductase	5.59	*HOXB9*	homeobox B9	2.78
*SLC3A1*	solute carrier family 3, member 1	6.12	*SPIN3*	spindlin family, member 3	2.89
*THG1L*	tRNA-histidine guanylyltransferase 1-like	6.20	*IGF2*	insulin-like growth factor 2	2.94
*WNT7A*	wingless-type MMTV integration site family, member 7A	7.15	*FOLR1*	folate receptor 1 (adult)	2.98
*TNSF10*	tumor necrosis factor (ligand) superfamily, member 10	7.92	*PAQR8*	progestin and adipoQ receptor family member VIII	3.04

**Table 3:** After DSCAM-AS1 knockdown (KD) by >90% in endometrial cancer (HEC-1B) cells, the regulated genes (at least 2.5-fold change) were first annotated to the Gene Ontology (GO) category “Biological processes” using the software GOTermFinder (Version 22.9.2021) (https://go.princeton.edu/cgi-bin/GOTermFinder).

**Gene Ontology Annotation of Genes Downregulated after DSCAM-AS1 Knockdown in EC cells**		
**Gene Ontology Category** **“Biological Processes”** **(Activated by *DSCAM-AS1*)**	**Corrected** ***p*-Value**	**Downregulated Genes after DSCAM-AS1 KD (Cut-Off: 2.5-Fold), Annotated to the GO Terms**
positive regulation of cell migration GO: 0030335	3.61 × 10^−16^	*CLDN4, MAP2K3, PLP1, DOCK1, PLAU, BDKRB1, NOTCH1, CYR61, C10orf54, RIN2, ANXA3, PTK2, EDN2, TJP1, IFNG, PTN, SERPINE1*
negative regulation of cell death GO:0060548	1.62 × 10^−6^	*NOTCH1, UNC5B, HMGA2, PTK2, TJP1, ZFPM2, MECP2, GREM1, SERPINE1, PROK2, CYR61, CD34*
positive regulation of angiogenesis GO:0045766	1.30 × 10^−6^	*BMPER, GREM1, SERPINE1, ANXA3, HMGA2, TJP1, CD34*
negative regulation of apoptotic process GO:0043066	9.46 × 10^−5^	*NOTCH1, UNC5B, HMGA2, PTK2, TJP1, MECP2, GREM1, SERPINE1, PROK2, CYR61*
positive regulation of cell proliferation GO:0008284	0.00014	*NOTCH1, HMGA2, PTK2, FOSL1, GREM1, EGR1*
**Gene Ontology Annotation of Genes Upregulated after DSCAM-AS1 Knockdown in EC cells**		
**Gene Ontology Category** **“Biological Processes”** **(Inhibited by *DSCAM-AS1*)**	**Corrected** ***p*-Value**	**Upregulated Genes after DSCAM-AS1 KD (Cut-Off: 2.5-Fold), Annotated to the GO Terms**
positive regulation of developmental process GO:0051094	8.59 × 10^−14^	*SEMA4A, IRX3, DKK1, SMAD7, SEMA4D, LINGO2, C5AR1, IGF1R, PLXNB2, CAMK2B, WNT7A, RELN, PLXNB1, FN1, TGFBR1, INSR, SLITRK5*
positive regulation of apoptotic process GO:0043065	3.87 × 10^−8^	*TNFSF10, HTRA1, SKIL, CYP1B1, BMP4, BMP2, TGFBR1, DKKL1, FOXO3, ZC3H12A, IGFBP3*
positive regulation of cell junction assembly GO:1901890	1.24 × 10^−8^	*SEMA4A, IRX3, ACE2, WNT7A, SEMA4D, EPB41L5, LINGO2, SLITRK5*
positive regulation of cell development GO:0010720	2.04 × 10^−6^	*PLXNB2, CAMK2B, SMAD7, RELN, SEMA4D, PLXNB1, EPHA4, FN1, CX3CL1*
positive regulation of cell differentiation GO:0045597	1.34 × 10^−5^	*IRX3, DKK1, PLXNB2, CAMK2B, SMAD7, RELN, SEMA4D, PLXBN1, EPHA4, FN1, CX3CL1, TGFBR1*

## Data Availability

Data will be provided on request.

## Supplementary Materials

The following supporting information can be downloaded at: https://www.mdpi.com/article/10.3390/biomedicines10071727/s1, Figure S1: Genes of the estrogen pathway associated with DSCAM-AS1 expression in EC.; Figure S2: Genes with equidirectional regulation after DSCAM knockdown in HEC-1B and RL95-2 cells, indicated is their association with OS. Figure S3: Genes being most significantly downregulated after DSCAM-AS1 knockdown in HEC-1B cells, shown is their association with OS. Figure S4: Genes being strongly downregulated after DSCAM-AS1 knockdown in EC cells, shown is their correlation with DSCAM-AS1 in endometrial adenocarcinoma tissues. Table S1: Sequences of primers used for RT-qPCR. Table S2: Gene Set Enrichment Analysis (GSEA) of genes up- or downregulated upon DSCAM-AS1 knockdown in HEC-1B cells [30]. Supplementary file S1: Genes correlated with DSCAM-AS1 (R-value cut off 0.4) in endometrial adenocarcinoma (using open-source dataset “Tumor Endometrium—EXPO—209—MAS5.0—u133p2”, GEO ID: GSE2109.
