# Supplementary material for: DSCAM-AS1 Long Non-Coding RNA Exerts Oncogenic Functions in Endometrial Adenocarcinoma via Activation of a Tumor-Promoting Transcriptome Profile
Source: Biomedicines. 2022 Jul 18;10(7):1727. doi: 10.3390/biomedicines10071727 (PMC9313190; doi:10.3390/biomedicines10071727)
Supplement: Supplementary file 1 [file biomedicines-10-01727-s001.zip › suplementary materials.pdf]

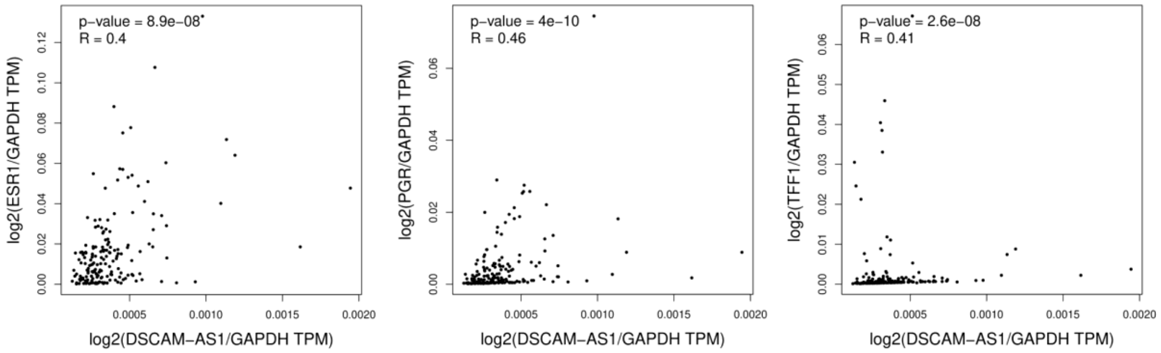

Figure S1: Genes of the estrogen pathway associated with DSCAM-AS1 expression in EC.

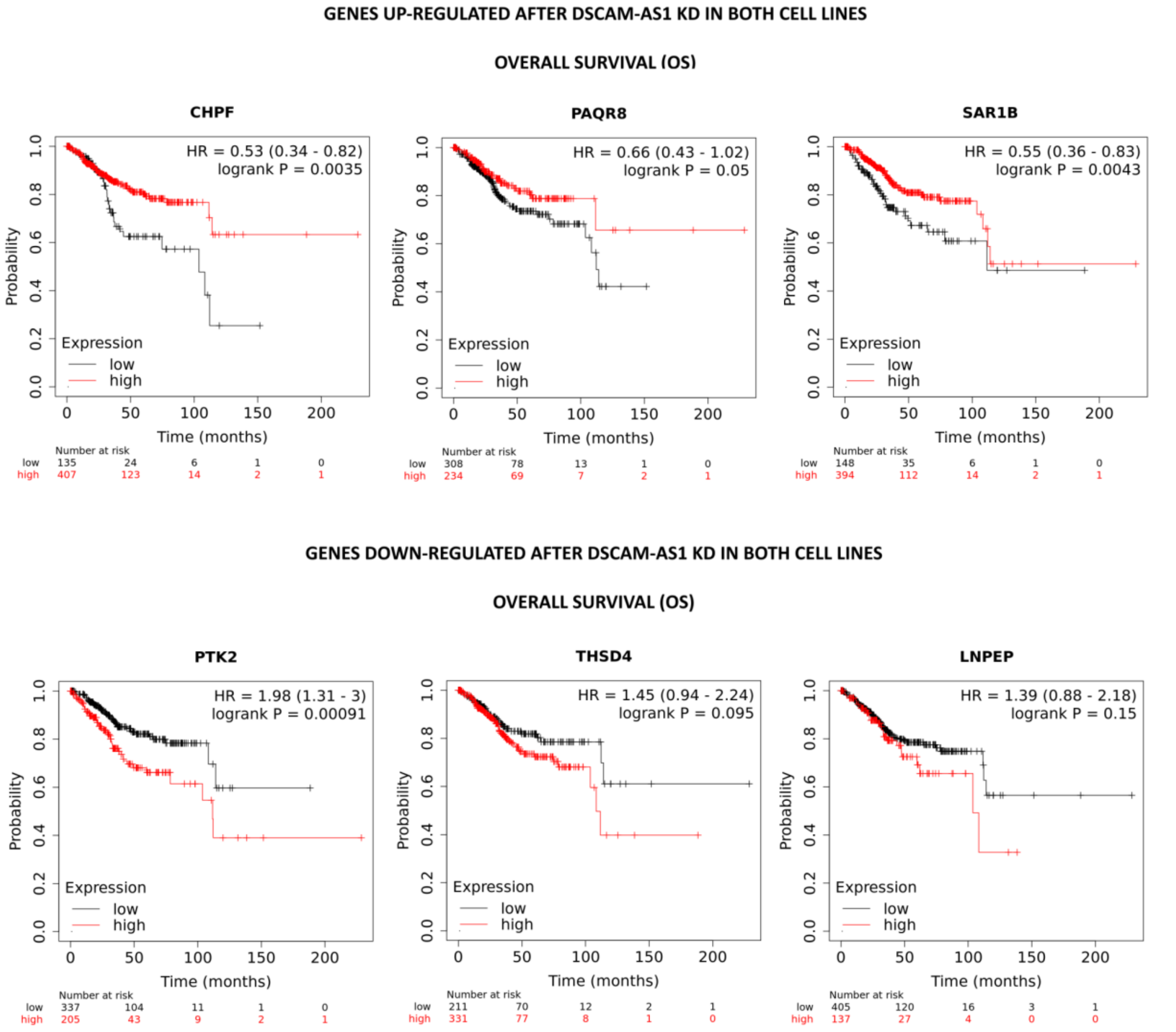

Figure S2: Genes with equidirectional regulation after DSCAM knockdown in HEC-1B and RL95-2 cells, indicated is their association with OS.

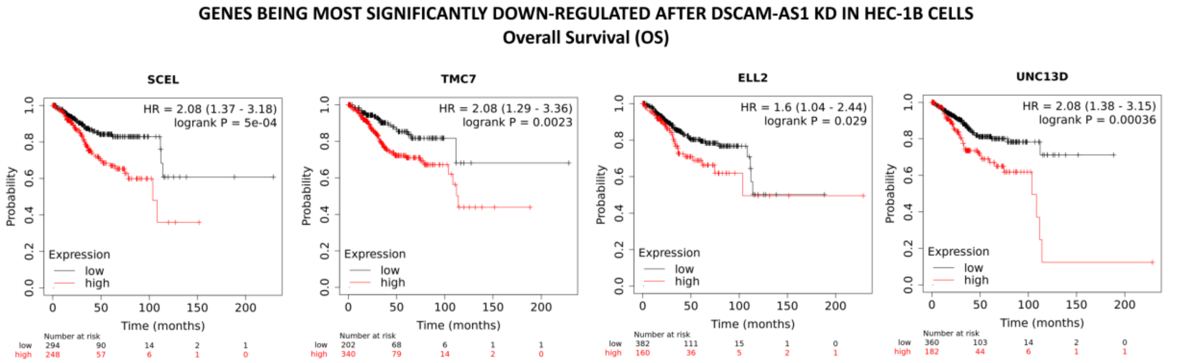

Figure S3: Genes being most significantly downregulated after DSCAM-AS1 knockdown in HEC-1B cells, shown is their association with OS.

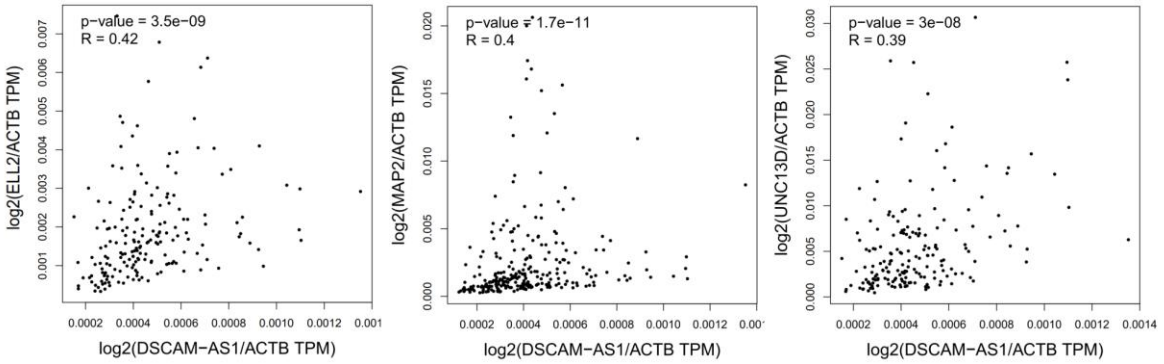

Figure S4: Genes being strongly downregulated after DSCAM-AS1 knockdown in EC cells, shown is their correlation with DSCAM-AS1 in endometrial adenocarcinoma tissues.
