# Supplementary material for: DSCAM-AS1 Long Non-Coding RNA Exerts Oncogenic Functions in Endometrial Adenocarcinoma via Activation of a Tumor-Promoting Transcriptome Profile
Source: Biomedicines. 2022 Jul 18;10(7):1727. doi: 10.3390/biomedicines10071727 (PMC9313190; doi:10.3390/biomedicines10071727)
Supplement: Supplementary file 1 [file biomedicines-10-01727-s001.zip › Table S1.pdf]

Supplemental Table S1. Sequences of primers used for RT-qPCR

Gene	Primer (5´-3´)	Amplicon [bp]
**DSCAM-AS1**	CTGCAGGGCTGACGTGG<br>GTCTGGTTGGCTTTCCCGAT	114
**ESR1**	CACATGAGTAACAAAGGCATGG<br>ATGAAGTAGAGCCCGCAGTG	181
**SCEL**	CGAGCAGAAGAAGTGAAGACCT<br>ATTATCAAGGCTCTGGCCCC	149
**PLAU**	CCAGGGTCCACCTGTCCC<br>CATTGCTGCCTTTGGAGTCG	153
**WNT7A**	TCGGGACTATGAACCGGAAA<br>TGAGGAGAAGCCACCGATCC	92
**TNFSF10**	TCCTCAGAGAGTAGCAGCTCA<br>TTGGAGTTTGGAGAAGACAATGTG	72
**ACTB**	CTTCCTTCCTGGGCATGGAGT<br>CAGGAGGAGCAATGATCTTGATCTTC	210
