# Supplementary material for: DSCAM-AS1 Long Non-Coding RNA Exerts Oncogenic Functions in Endometrial Adenocarcinoma via Activation of a Tumor-Promoting Transcriptome Profile
Source: Biomedicines. 2022 Jul 18;10(7):1727. doi: 10.3390/biomedicines10071727 (PMC9313190; doi:10.3390/biomedicines10071727)
Supplement: Supplementary file 1 [file biomedicines-10-01727-s001.zip › Table S2 new.pdf]

Table S2: Gene Set Enrichment Analysis (GSEA) using MSigDB V7.5.1 of genes up- or down-regulated upon DSCAM-AS1 knockdown in HEC-1A cells by calculation of gene set overlaps. (http://www.gsea-msigdb.org/gsea/index.jsp) (FDR = false detection rate)

**Genes down-regulated upon DSCAM-AS1 knockdown: overlapping gene sets**				
Gene Set Name [# Genes (K)]	Description	# Genes in Overlap	p-value	FDR
KOINUMA_TARGETS_OF_SMAD2_OR_SMAD3 [845]	Genes with promoters occupied by SMAD2 or SMAD3 [GeneID=4087, 4088] in HaCaT cells (keratinocyte) according to a ChIP-chip analysis.	20	$4.38\ e^{-13}$	$1.41\ e^{-8}$
ANDERSEN_CHOLANGIOCARCINOMA_CLASS2 [175]	Genes overexpressed in cholangiocarcinoma class 2 associated with poor prognosis.	11	$3.87\ e^{-12}$	$6.22\ e^{-8}$
ONDER_CDH1_TARGETS_2_DN [473]	Genes down-regulated in HMLE cells (immortalized nontransformed mammary epithelium) after E-cadhedrin (CDH1) [GeneID=999] knockdown by RNAi.	14	$1.02\ e^{-10}$	$1.09\ e^{-6}$
CHARAFE_BREAST_CANCER_LUMINAL_VS_BASAL SAL_DN [456]	Genes down-regulated in luminal-like breast cancer cell lines compared to the basal-like ones.	12	$8.78\ e^{-9}$	$4.07\ e^{-5}$
GOBP_LOCOMOTION [1921]	Self-propelled movement of a cell or organism from one location to another.	22	$2.61\ e^{-8}$	$9.81\ e^{-5}$
ENK_UV_RESPONSE_EPIDERMIS_DN [513]	Genes down-regulated in epidermis after to UVB irradiation.	12	$3.2\ e^{-8}$	$1.03\ e^{-4}$
FORTSCHEGGER_PHF8_TARGETS_DN [783]	Genes down-regulated in HeLa cells (cervical carcinoma) upon knockdown of PHF8 [GeneID=23133] by RNAi.	14	$6.12\ e^{-8}$	$1.79\ e^{-4}$
KIM_WT1_TARGETS_12HR_DN [217]	Genes down-regulated in UB27 cells (osteosarcoma) at 12 hr after inducing the expression of a mutated form of WT1 [GeneID=7490].	8	$2.34\ e^{-7}$	$4.43\ e^{-4}$
PASINI_SUZ12_TARGETS_DN [316]	Genes down-regulated in ES (embryonic stem cells) with defficient SUZ12 [GeneID=23512].	9	$3.56\ e^{-7}$	$6.37\ e^{-4}$
GOBP_CELL_MIGRATION [1556]	The controlled self-propelled movement of a cell from one site to a destination guided by molecular cues. Cell migration is a central process in the development and maintenance of multicellular organisms.	18	$4.9\ e^{-7}$	$7.89\ e^{-4}$

Genes up-regulated upon DSCAM-AS1 knockdown: overlapping gene sets				
Gene Set Name [# Genes (K)]	Description	# Genes in Overlap	p-value	FDR
DOUGLAS_BMI1_TARGETS_UP [563]	Genes up-regulated in A4573 cells (Ewing's sarcoma, ESFT) after knockdown of BMI1 [GeneID=648] by RNAi.	33	$7.07\ e^{-26}$	$7.27\ e^{-22}$
MEISSNER_BRAIN_HCP_WITH_H3K4ME3_AND_H3_H3K27ME3 [1073]	Genes with high-CpG-density promoters (HCP) bearing histone H3 dimethylation at K4 (H3K4me2) and trimethylation at K27 (H3K27me3) in brain.	27	$2.87\ e^{-12}$	$1.47\ e^{-8}$
MIR217_5P [187]	Genes predicted to be targets of miRBase v22 microRNA hsa-miR-217-5p in miRDB v6.0 with MirTarget v4 prediction scores > 80 (high confidence targets).	13	$8.99\ e^{-12}$	$3.08\ e^{-8}$
MIR8485 [1034]	Genes predicted to be targets of miRBase v22 microRNA hsa-miR-8485 in miRDB v6.0 with MirTarget v4 prediction scores > 80 (high confidence targets).	24	$2.61\ e^{-10}$	$6.71\ e^{-7}$
WONG_ADULT_TISSUE_STEM_MODULE [721]	The 'adult tissue stem' module: genes coordinately up-regulated in a compendium of adult tissue stem cells.	20	$4.34\ e^{-10}$	$8.92\ e^{-7}$
ESC_V6.5_UP_LATE.V1_UP [182]	Genes up-regulated during late stages of differentiation of embryoid bodies from V6.5 embryonic stem cells.	11	$1.64\ e^{-9}$	$2.81\ e^{-6}$
MIR6807_3P [142]	Genes predicted to be targets of miRBase v22 microRNA hsa-miR-6807-3p in miRDB v6.0 with MirTarget v4 prediction scores > 80 (high confidence targets).	10	$2.1\ e^{-9}$	$2.81\ e^{-6}$
LEF1_UP.V1_DN [187]	Genes down-regulated in DLD1 cells (colon carcinoma) over-expressing LEF1 [Gene ID=51176].	11	$2.19\ e^{-9}$	$2.81\ e^{-6}$
MIR548AV_5P_MIR548K [307]	Genes predicted to be targets of miRBase v22 microRNA hsa-miR-548av-5p, hsa-miR-548k in miRDB v6.0 with MirTarget v4 prediction scores > 80 (high confidence targets).	13	$4.05\ e^{-9}$	$4.16\ e^{-6}$
MIR8054 [307]	Genes predicted to be targets of miRBase v22 microRNA hsa-miR-8054 in miRDB v6.0 with MirTarget v4 prediction scores > 80 (high confidence targets).	13	$4.05\ e^{-9}$	$4.16\ e^{-6}$
